# Large-Scale High-Accuracy and High-Efficiency Phase Plate Machining

**DOI:** 10.3390/nano14191563

**Published:** 2024-09-27

**Authors:** Guanhua Wang, Zhaoxiang Liu, Lvbin Song, Jianglin Guan, Wei Chen, Jian Liu, Jinming Chen, Min Wang, Ya Cheng

**Affiliations:** 1 State Key Laboratory of Precision Spectroscopy, East China Normal University, Shanghai 200062, China; 52210920028@stu.ecnu.edu.cn (G.W.); lbsong@stu.ecnu.edu.cn (L.S.); 51200920051@stu.ecnu.edu.cn (J.G.); 52210920027@stu.ecnu.edu.cn (J.L.); 2 The Extreme Optoelectromechanics Laboratory (XXL), School of Physics and Electronic Science, East China Normal University, Shanghai 200241, China; wchen@phy.ecnu.edu.cn (W.C.); jmchen@phy.ecnu.edu.cn (J.C.); mwang@phy.ecnu.edu.cn (M.W.); 3 State Key Laboratory of High Field Laser Physics and CAS Center for Excellence in Ultra-Intense Laser Science, Shanghai Institute of Optics and Fine Mechanics (SIOM), Chinese Academy of Sciences (CAS), Shanghai 201800, China; 4 Collaborative Innovation Center of Extreme Optics, Shanxi University, Taiyuan 030006, China; 5 Collaborative Innovation Center of Light Manipulations and Applications, Shandong Normal University, Jinan 250358, China; 6 Hefei National Laboratory, Hefei 230088, China; 7 Shanghai Research Center for Quantum Sciences, Shanghai 201315, China

**Keywords:** diffractive optical elements, photolithography, femtosecond laser micromachining, Fresnel phase plate

## Abstract

In this paper, multifunctional, multilevel phase plates of quartz substrate were efficiently prepared by using a newly developed polygon scanner-based femtosecond laser photolithography system combined with inductively coupled discharge plasma reactive-ion etching (ICP-RIE) technology. The femtosecond laser photolithography system can achieve a scanning speed of 5 m/s and a preparation efficiency of 15 cm^2^/h while ensuring an overlay alignment accuracy of less than 100 nm and a writing resolution of 500 nm. The ICP-RIE technology can control the etching depth error within ±5 nm and the mask-to-mask edge error is less than 1 μm. An 8-level Fresnel lens phase plate with a focal length of 20 mm and an 8-level Fresnel axicon phase plate with a cone angle of 5° were demonstrated. The diffraction efficiency was greater than 93%, and their performance was tested for focusing and glass cutting, respectively. Combined with the high-speed femtosecond laser photolithography system’s infinite field-of-view (IFOV) processing capability, the one-time direct writing preparation of phase plate masks of different sizes was realized on a 6-inch wafer. This is expected to reduce the production cost of quartz substrate diffractive optical elements and promote their customized mass production.

## 1. Introduction

The manipulation of laser light fields is increasingly important in scientific research and industrial production. Scientists have invented a variety of technologies to manipulate laser light fields, such as spatial light modulators [1,2], slit/grating shaping technology [3,4], meta-surface technology [5,6], various diffractive optical elements (DOEs) [7,8], and so on.

The DOEs have attracted much attention due to their small size, high flexibility, compact structure, and mass production in recent decades. The main means of achieving this is to change the distribution of the diffracted light field by changing the spatial phase distribution of the light field to accomplish various functions. Since the phase change is at the wavelength scale, the thickness of the optical element can be small, which is very conducive to constructing new optical systems. Currently, diffractive optical elements have been widely used in beam shaping [9,10,11], beam splitting [12,13], various structured light applications [14,15,16], and so on.

Usually, fused silica and liquid crystal polymers are the most commonly used substrate of the DOEs. Relatively, the fused silica-based DOEs can simplify the optical path and have the potential for use in high-power systems due to their high damage threshold. So far, the fused silica-based DOEs can be made through photolithography techniques adapted from the microelectronics industry [13], electron beam lithography (EBL) [17], focus ion beams [6], laser-induced microplasma fabrication (LIMP) [18], laser direct writing [19,20,21], etc. With the support of the microelectronics industry, the lithographic technique can make a perfect DOE, but its process is complicated, and the cost is very high. EBL cost is relatively low, but its exposure efficiency is limited, and the LIMP technology is insufficient in lateral precision. Generally, laser direct writing technology is compatible with cost, efficiency, and accuracy.

Ultrafast lasers have been widely used in micro–nano processing due to their excellent performance while interacting with materials, such as precision cutting [9] and micro–nano structure preparation [21,22]. However, traditional ultrafast laser direct writing technology is mainly based on low-speed single-point scanning, which makes it difficult to meet the industrial needs of mass production. We have made improvements to the optical system in the early stage [23], using a high-speed rotating polygon mirror scanning system combined with a three-dimensional precision motion control platform to achieve infinite field-of-view (IFOV) processing capabilities, which have been promoted in the preparation of large-scale thin-film lithium niobate on insulators (LNOI) photonic devices and multifunctional phase plates.

Here, we propose a high-speed femtosecond laser processing system combined with inductively coupled discharge plasma reactive-ion etching (ICP-RIE) technology to efficiently machine multifunctional, multilevel phase plates, which perfectly solves the incompatibility problem between the lateral processing accuracy and efficiency and the longitudinal resolution of a femtosecond laser. Through the upgraded high-speed rotating mirror scanning system, the lateral writing resolution is set to 500 nm with a scanning speed reaching 5 m/s, much higher than the traditional single-point direct writing with the highest motion speed of 500 mm/s. The longitudinal etching depth error can be controlled within ±5 nm, which is much smaller than the femtosecond laser size. The experiment demonstrates an 8-level Fresnel lens phase plate with a focus length of 20 mm and an 8-level Fresnel axicon phase plate with a cone angle of 5°, with a diffraction efficiency higher than 93%. This technology greatly improves the preparation efficiency while ensuring the diffraction efficiency of the phase plate. It can potentially assist in the mass production of large-scale high-accuracy phase plates, which will be widely used in manufacturing and scientific research.

## 2. Experimental Design and Methods

### 2.1. Femtosecond Laser Photolithography System

**Figure 1:**
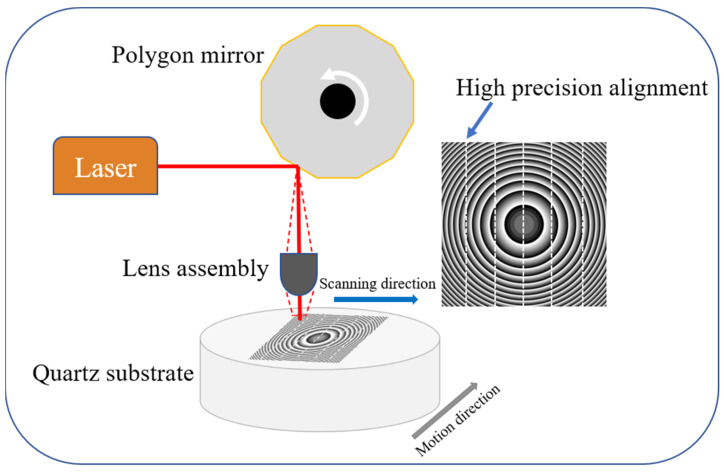
High-precision infinite field-of-view femtosecond laser direct writing system.

The self-developed high-precision and high-resolution IFOV femtosecond laser photolithography system mainly consists of a femtosecond laser, a polygon mirror scanner, and a precision motion platform, and the synchronization and precise control of the three are realized through the fully self-developed FPGA hardware module and software. The surface flatness error is calibrated using real-time autofocus on the sample surface. The principle of optical path transmission is shown in Figure 1. A femtosecond laser with a wavelength of 1030 nm (Satsuma, Amplitude Inc., Pessac, France) is transmitted to a polygon mirror scanner (SB12, Lincoln Laser Inc., Phoenix, AZ, USA). The femtosecond laser system provides a femtosecond laser with an average power of up to 10 W, a repetition rate of 40 MHz, and a pulse duration of 350 fs. The high-performance polygon mirror scanner rotates at high speed in a fixed direction and the laser repeatedly scans (>3000 lines/s) along the fixed direction within the scanning angle. The scanned light field is focused on the surface of the sample by the optical system to form a linear light spot distribution.

The control system analyzes and processes the signal within 25 ns, converts the mask pattern into a laser control signal, and accurately controls the switching light in each scanning line. At the same time, the motion platform moves perpendicular to the scanning direction to form a strip-shaped scanning area, and high-precision stitching calibration and error compensation are performed on each scanning area. Compared with the previous system, this system increases the direct writing area and theoretically can realize one-time direct writing of 12-inch wafer samples. In addition, the scanning speed and resolution have been optimized, and the scanning speed of the motion platform is up to 5 m/s while ensuring the engraving alignment accuracy error is less than 100 nm and the writing resolution is 500 nm; the preparation efficiency reaches 15 cm^2^/h.

### 2.2. Multilevel Phase Mask Design Method

The diffractive optical element can be prepared using liquid crystal polymers or through etching quartz substrates to print phase information onto the element board. There are various etching methods for quartz substrates, including directly forming, or preparation using mask patterns with the help of a photolithography process. Ideally, a continuous phase distribution like a Fresnel zone plate can achieve 100% diffraction efficiency, but it is hard to fabricate. Currently, the most common method is to quantify the continuous phase distribution into discrete phase order, and the phase plate of order 2^n^ can be prepared by n times of mask writing. Theoretically, the designed 8-level phase plate can achieve 94.96% diffraction efficiency [24], which requires mask preparation and etching to be carried out 3 times.

For multilevel diffractive optical elements, the total depth h corresponding to phase  2π satisfies the following [25]:

(1) $$h\left[\frac{2\pi n_{1}}{\lambda }-\frac{2\pi n_{0}}{\lambda }\right]=2\pi$$

where *n*_1_ is the refractive index of the medium, *n*_0_ is the refractive index of air, the corresponding depth *h*_1_ of each level satisfies *h*_1_ *= h*/*L*, and *L* is the number of levels.

Here, the high-accuracy and high-efficiency diffractive optical devices are introduced through the preparation and characterization of the multilevel Fresnel lens and the multilevel Fresnel axicon. The transmission function is a complex exponential function with unit amplitude for the Fresnel lens. In the paraxial approximation, the transmission function can be expressed as follows [26]:

(2) $$t_{lens}\left(x,y;\lambda \right)=exp\;[\frac{-i\pi (x^{2}+y^{2})}{\lambda f}]$$

*f* is the focal length of the lens, *x*, *y* are the coordinates in the lens plane, *λ* is the wavelength of the light, and the spot size *d* of Gaussian light focused by the lens can be written as follows:

(3) $$d=\frac{4M^{2}\lambda f}{\pi D}$$

where the *M*^2^ factor is the beam quality factor of the laser, and *D* is the diameter of the incident laser.

The axicon is determined by angle α, and the Gaussian light passing through the axicon will form a non-diffractive transmission Bessel beam, which has great advantages in laser cutting, optical tweezers, and imaging applications. The transmission function of the axicon can be described as follows [27]:

(4) $$T_{ax}\left(r\right)=exp\;(i\beta r)$$

where *β* is the linear slope value.

(5) β=2π(n−1)α/λ

*λ* is the wavelength of the laser, *α* is the angle of the prism, *n* is the refractive index, $r=\sqrt{x^{2}+y^{2}}$.

**Figure 2:**
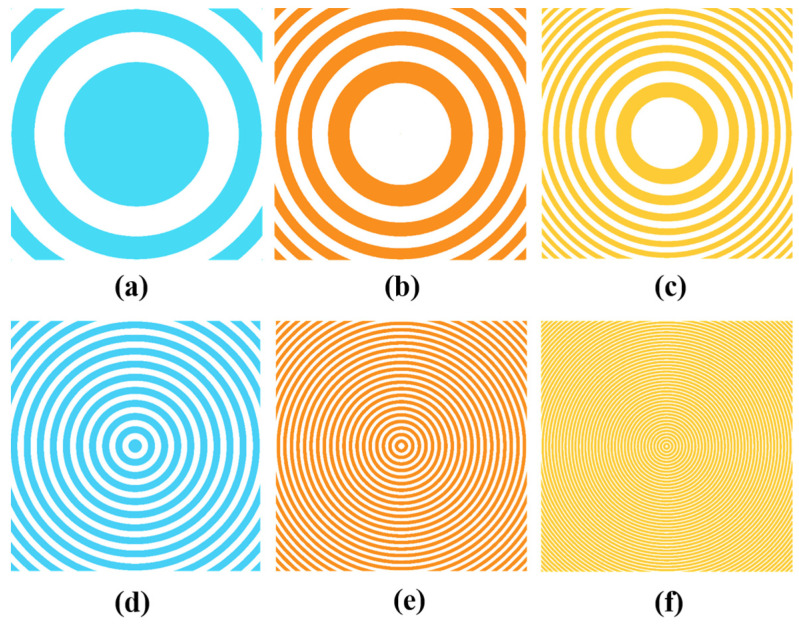
(**a**–**f**) correspond to the mask patterns M1, M2, and M3 in three photolithographic processes of the Fresnel lens and Fresnel axicon, respectively.

By extracting the phase components of the transmission Functions (2) and (4) and using formula (1) to design three photolithography masks, an 8-level quantized phase plate can be designed. As can be seen, Figure 2a–f correspond to the mask patterns M1, M2, and M3 in three photolithographic processes of the Fresnel lens and Fresnel axicon, respectively. For the quartz substrate phase plate with a light source wavelength of 1030 nm, the corresponding lithography depth of each mask is $h_{M1}$ = 1144.4 nm, $h_{M2}$ = 572.2 nm, $h_{M3}$ = 286.1 nm, and each mask pattern corresponds to 2-level, 4-level, and 8-level phase plates, respectively, after step-by-step processing.

### 2.3. Cleaning and Etching Process

Each step of fabrication of the 8-level phase plates requires going through Cr film coating, femtosecond laser direct writing, cleaning, ICP-RIE, and other processes.

**Figure 3:**
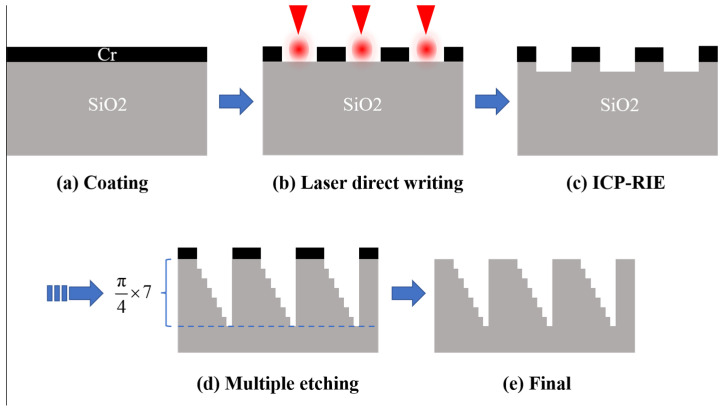
A flow chart of the femtosecond laser photolithography-assisted ICP-RIE in fabricating multilevel phase plates.

As shown in Figure 3, a 100 nm Cr metal film is first coated on the surface of the SiO_2_ substrate (Figure 3a). Before that, the cleaning process is used to remove the ionic and metallic contaminants as well as the native oxide and other associated organic contaminants. Thus, the wafers were immersed into the mixture of (H_2_SO_4_: H_2_O_2_) in the ratio of 2:1 (by volume) at the temperature of 120 °C for a duration of 10 min, and then placed in deionized water and sonicated at 80 °C for 5 min. Ultrasonic shaking was performed for 10 min for the mixture of (H_2_O: NH_4_OH: H_2_O_2_) in the ratio of 5:1:1 to remove particles. Finally, the samples were rinsed with deionized water and blow-dried with nitrogen gas (N_2_). After coating, the mask pattern was printed on the Cr film using the polygon scanner-based femtosecond photolithography system (Figure 3b) by ablating the Cr metal film with a femtosecond laser power of 300 mW, a repetition rate of 10 MHz, a pulse duration of 350 fs, and a writing speed of 5 m/s. And then SiO_2_ was etched using ICP-RIE technology (Figure 3c). For the ICP-RIE (PlasmaPro^®^100, Oxford Inc., London, UK), the main etchant used in this work is C_4_F_8_/O_2_ at the flow rate of 40/20 SCCM; the inductively coupled plasma radio frequency (ICP RF): 2000 W, Table RF: 200 W, process pressure 10 mTorr. The etching rate was approximately 3.2 nm/s to 4 nm/s. After multiple etching compensations to optimize the depth, each stage of the etching process was controlled within the error range ±5 nm. After etching, the surface Cr film was corroded off using Chromium etchant. The same operation is repeated until the 8-level phase plate is formed.

## 3. Fabrication Characterization and Functional Demonstration

### 3.1. Fabrication Characterization

**Figure 4:**
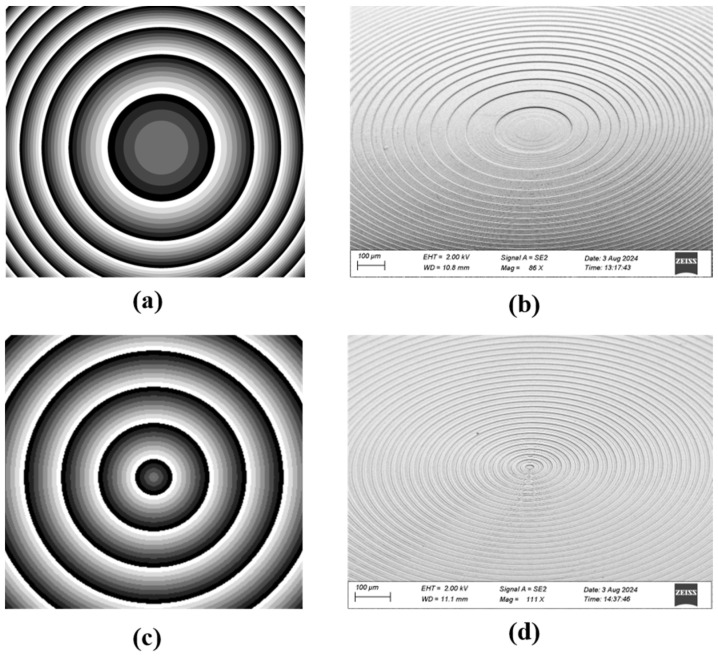
Phase design diagram (**a**) and SEM image (**b**) of 8-level Fresnel lens; phase diagram (**c**) and SEM image (**d**) of 8-level Fresnel axicon.

We characterized the morphology and structure of the prepared phase plates. The phase design diagrams of the 8-level Fresnel lens and 8-level Fresnel axicon, as well as the scanning electron microscope (SEM) (Gemini 450, Zeiss Inc., Oberkochen, Germany) images of the samples, are shown in Figure 4. The eight grayscale values in the phase diagram (Figure 4a,c) correspond to the eight phase values discretized. Figure 4b,d show the SEM images with a sample tilt angle of 40° of the Fresnel lens and Fresnel axicon, respectively. The surface roughness of the sample is relatively perfect, and the edge error of each level is less than 1 μm, which is limited by the processing resolution of the system.

**Figure 5:**
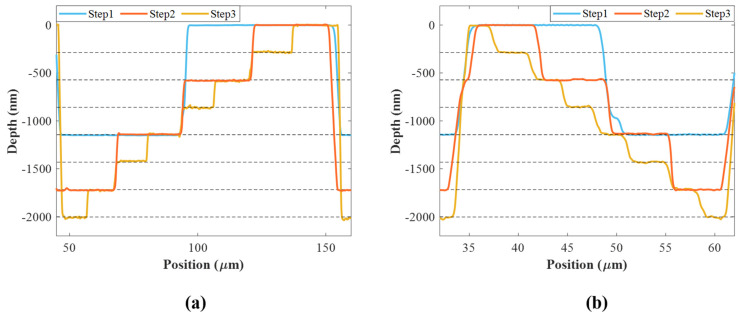
The partial depth variation curve of the three times mask preparations of the 8-level Fresnel lens (**a**) and Fresnel axicon (**b**). Steps 1 to 3 correspond to the mask preparation results of M1 to M3, while black dashed lines indicate the theoretical depth for each level.

To accurately control the etching depth, we have characterized the etching depth of the mask during the ICP-RIE process. Figure 5 shows the partial depth variation curve of the mask preparations that were carried out three times and measured using the profilometer, from the deepest point to the surface corresponding to the phase change from −π to π. The measured height difference between the minimum and maximum levels is 2037 nm. Since the theoretical value is 2003 nm, the relative error after fabrication is only 1.75%. The total depth error of the seven levels is 34 nm, and the average error to each level is only 5 nm. The curves from Step 1 to Step 3 correspond to the mask M1 to M3 photolithographic processes depth after etching. We compare the actual depth of each level with the theoretical value, and each level’s etching process can be controlled within the error range of ±5 nm. For the Fresnel lens (Figure 4a and Figure 5a), its mask patterns gradually densify from the center to the edge, so the width of each level is different. For the 8-level Fresnel axicon with the angle of prism α = 5° (Figure 4c and Figure 5b), the width of each level is the same, and after the third mask preparation, the width is less than 4 μm. Due to the size limitation of the profilometer probe, there is an error in the position direction, resulting in a slight misalignment compared with the results of the previous two measurements.

**Figure 6:**
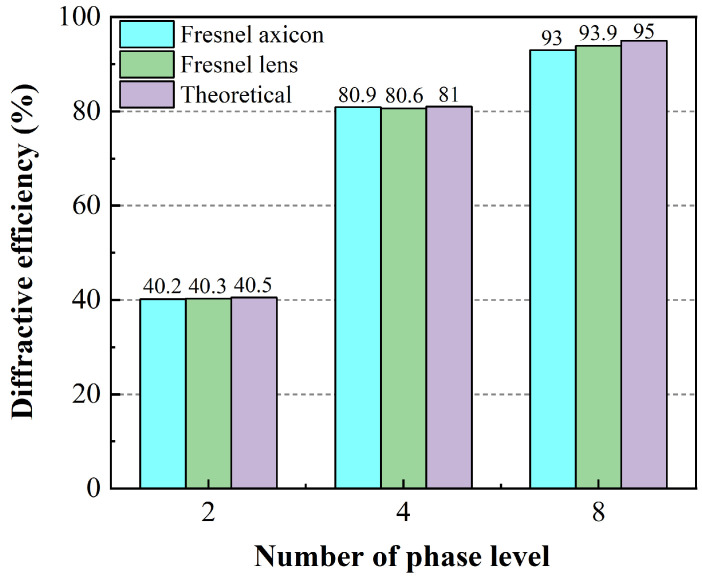
Graph of the diffraction efficiency as a function of the number of phase levels. The blue and green bars are the experimental diffraction efficiency of the Fresnel axicon and the Fresnel lens, respectively. The purple bars are the theoretical diffraction efficiency.

Meanwhile, we measured the phase plates’ effective diffraction power and total power using a femtosecond laser with a wavelength of 1030 nm (Satsuma, Amplitude Inc., Pessac, France) and a power meter (Nova II, Ophir Inc., Jerusalem, Israel) after each mask machining and calculated the diffraction efficiency. We used an aperture to cut the effective diffraction area, filter out high-order modes, and measure the effective diffraction power. The power meter was closely attached to the phase plates to measure the total power, and the diffraction efficiency of the phase plates was calculated by dividing the effective diffraction power by the total power. As shown in Figure 6, with the increase in phase level, the diffraction efficiency increases significantly, and the diffraction efficiency measured by the phase plates is very close to the theoretical value.

### 3.2. Functional Demonstrating

**Figure 7:**
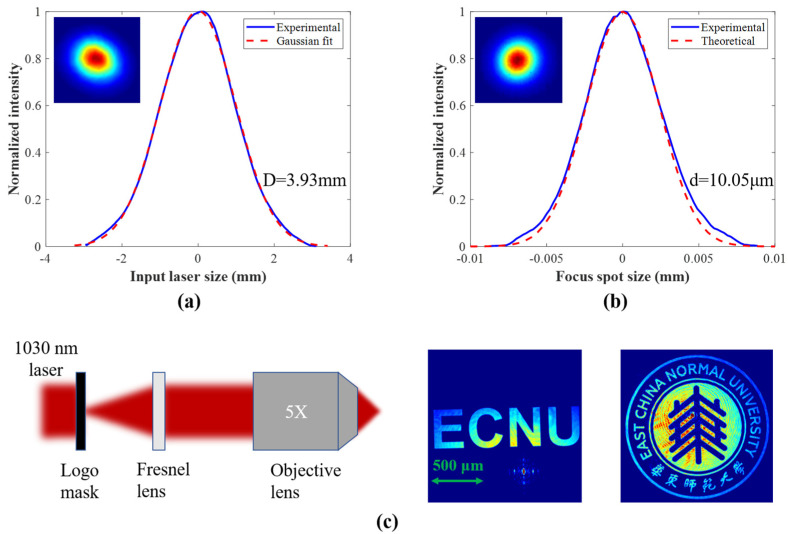
(**a**) The input collimated laser beam profile with a wavelength of 1030 nm; (**b**) focused spot profile of the Fresnel lens; (**c**) schematic diagram of Fresnel lens imaging (**left**) and the imaging patterns of the masks captured using CCD camera (**right**).

We validated the focusing and imaging capabilities of the Fresnel lens fabricated using the femtosecond laser photolithography system with a 1030 nm laser source. Figure 7a shows the profile of the incident laser measured using a charge-coupled device (CCD) camera, where the solid blue line is the measured beam profile, and the dashed red line is the Gaussian-fitted beam profile. The 1/e^2^ diameter is measured at D = 3.93 mm. After focusing the laser through the Fresnel lens, a CCD camera is used to measure the size of the focused spot. Here, a 4-f system is used to magnify and image the focused spot; the focused beam 1/e^2^ diameter is d = 10.05 μm (Figure 7b), which is almost consistent with the theory. We used a collimated laser with a wavelength of 1030 nm to illuminate the logo mask patterns and these were collected using the Fresnel lens to test the imaging capability. The schematic diagram of the Fresnel lens imaging experiment is shown on the left of Figure 7c, and the imaging patterns of the masks were collected using a CCD camera as displayed on the right, including micro-letters and micro-patterns, with clear imaging edge and distinguishable microstructures. These results confirm the excellent focusing/imaging quality of the Fresnel lenses.

**Figure 8:**
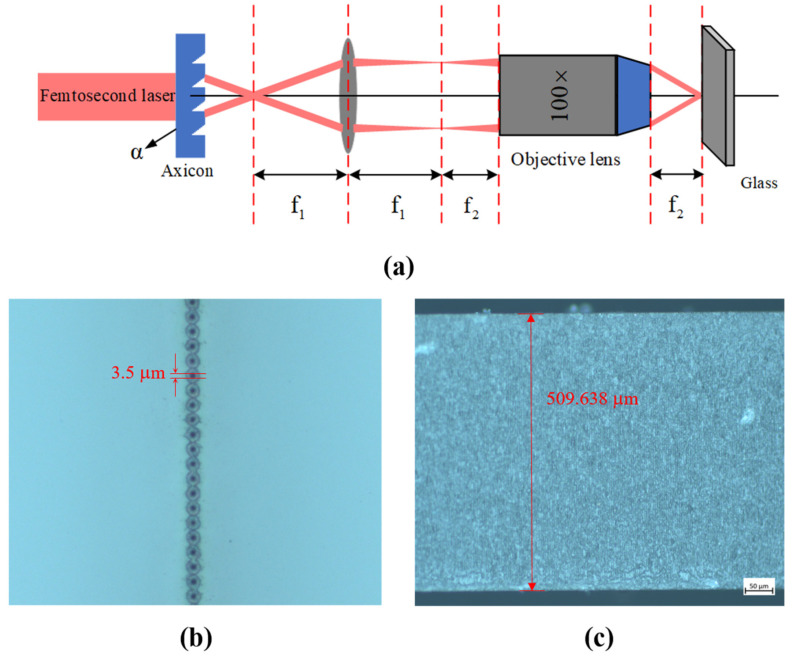
(**a**) Schematic diagram of the cutting experiment; (**b**) microscope image of the glass surface after laser processing; (**c**) microscope image of the glass cutting section.

The Fresnel axicon is directly applied to cutting glass samples. Figure 8a is a schematic diagram of the cutting experiment. Figure 8b is the microscope image of the glass surface after laser processing, with a dense array of dots representing the laser processing area, and each spot size is approximately 3.5 μm. Figure 8c is the microscope image of a glass cutting section with a thickness of 0.5 mm after splitting. The micro-cracks are evenly distributed in the transverse area of the laser beam, and the glass can easily split with a smooth cutting section.

### 3.3. Efficient Preparation of Large-Scale DOEs

**Figure 9:**
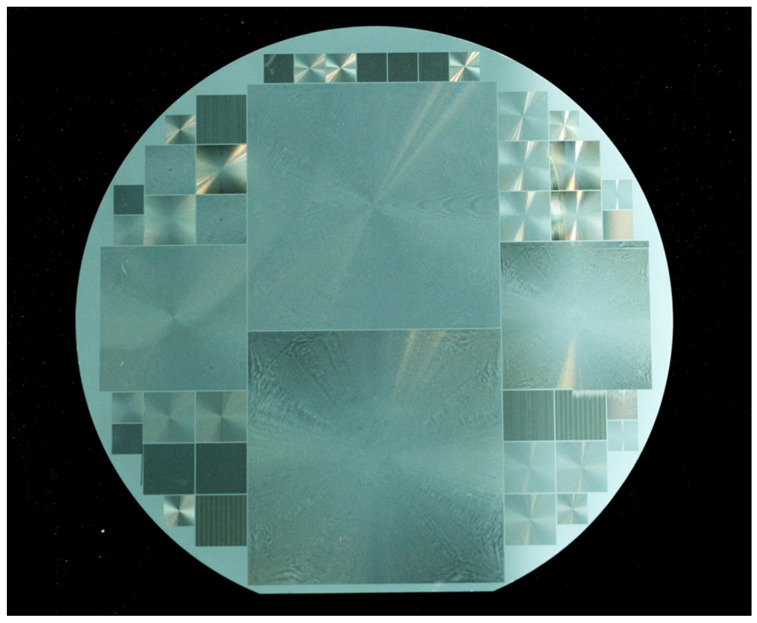
Large-scale diffractive optical elements fabricated on a 6-inch quartz wafer.

In addition, we used this ultra-high speed and high-resolution polygon scanner-based femtosecond laser photolithography system to achieve the one-time direct writing preparation of phase plate masks of different sizes on the surface of a 6-inch quartz wafer, including diffractive optical elements such as the Fresnel lens, axicon, vortex lens, grating, and checkerboard grating with various parameters, as shown in Figure 9.

## 4. Conclusions

To conclude, multifunctional 8-level quartz substrate phase plates are efficiently prepared using a newly developed polygon scanner-based femtosecond laser photolithography system combined with the ICP-RIE technology. The high-precision femtosecond laser photolithography system has excellent mask preparation efficiency and processing accuracy, which can achieve a scanning speed of 5 m/s and a preparation efficiency of 15 cm^2^/h while ensuring the overlay alignment accuracy of less than 100 nm and a writing resolution of 500 nm. The etching error of each level can be controlled within a range of ±5 nm. The 8-level Fresnel lens and 8-level Fresnel axicon have been successfully prepared, with diffraction efficiency reaching 93%, and their performance in focusing and glass cutting is demonstrated. With the help of this processing method, the efficient preparation of complex optical diffraction elements can be achieved, and the one-time direct writing preparation of phase plate masks of different sizes was realized on a 6-inch wafer. In the future, the phase plates’ phase level can be increased to 16 or higher for special applications to improve the diffraction efficiency. This fabrication technology can reduce the fabrication cost of DOEs on quartz substrate and support customized mass production preparation, which will greatly reduce the design cycle of devices in scientific research and the equipment development costs in the industry.

## Data Availability

Data underlying the results presented in this paper are not publicly available at this time but may be obtained from the authors upon reasonable request.
